# Effects of Flavonoids in *Lysimachia clethroides* Duby on the Activities of Cytochrome P450 CYP2E1 and CYP3A4 in Rat Liver Microsomes

**DOI:** 10.3390/molecules21060738

**Published:** 2016-06-14

**Authors:** Zhi-Juan Zhang, Zhao-Yang Xia, Jin-Mei Wang, Xue-Ting Song, Jin-Feng Wei, Wen-Yi Kang

**Affiliations:** 1 Institute of Chinese Materia Medica, Henan University, Kaifeng 475004, China; 13101788597@163.com (Z.-J.Z.); lancerevolution@foxmail.com (Z.-Y.X.); wangjinmeiscp@126.com (J.-M.W.); 2 Kaifeng Key Laboratory of Functional Components in Health Food, Kaifeng 475004, China; 3 Minsheng College, Henan University, Kaifeng 475004, China; 15537870395@163.com

**Keywords:** *Lysimachia clethroides* Duby, flavonoids, HPLC, CYP2E1, CYP3A4, liver microsomes

## Abstract

Incubation systems were established to investigate the effects of quercetin, kaempferol, isoquercitrin and astragalin in *Lysimachia clethroides* Duby on the activities of CYP2E1 and CYP3A4 in rat liver microsomes *in vitro*. Probe substrates of 4-nitrophenol and testosterone as well as flavonoids at different concentrations were added to the incubation systems. After incubation, a validated high performance liquid chromatography (HPLC) method was applied to separate and determine the relevant metabolites. The results suggested that kaempferol exhibited a weak inhibition of CYP2E1 activity with an IC_50_ of 60.26 ± 2.54 μM, while quercetin and kaempferol caused a moderate inhibition of CYP3A4 activity with IC_50_ values of 18.77 ± 1.69 μM and 32.65 ± 1.32 μM, respectively. Isoquercitrin and astragalin had no effects on the activities of either CYP2E1 or CYP3A4. It could be speculated from these results that the inhibitory effects of quercetin and kaempferol on the activities of CYP2E1 and CYP3A4 could be the mechanisms underlying the hepatoprotective effects of *L. clethroides*.

## 1. Introduction

*Lysimachia clethroides* Duby (*L. clethroides*), belonging to the family Primulaceae, is found in mild regions of northeast China, the southwest and other eastern provinces [1]. Phytochemical research has showed that flavonoids and glycosides, triterpenes and organic acids are the main compounds in *L. clethroides* [2,3,4,5]. Pharmacological investigation showed that *L. clethroides* possesses antitumor [6,7], antioxidant [8], hypoglycemic [9] and hepatoprotective [10] effects. The flavonoids are a group of the most important active compounds in *L. clethroides*.

Glutamic pyruvic transaminase (GPT) and glutamic oxaloacetic transaminase (GOT) are the main non-specific functional enzymes in the liver [11,12], their activities reflect the degree of hepatocyte damage to a certain extent. Malondialdehyde (MDA) and superoxide dismutase (SOD) are the key indicators of the severity of lipid peroxidation [13]. Our previous pharmacodynamic studies showed that the levels of both GOT and GPT in serum in response to carbon tetrachloride (CCl_4_)-induced acute liver injury in mice were significantly decreased by intragastric administration of extracts of *L. clethroides*, and that the content of MDA in liver was significantly decreased while the level of SOD was significantly increased *in vivo* [14]. These results indicated that *L. clethroides* had hepatoprotective effect on CCl_4_-induced acute liver injury and its antioxidant effect was the one of the mechanisms for the hepatoprotective effect of *L. clethroides.* However, the hepatoprotective mechanisms for the active compounds in *L. clethroides* are complex and no studies have been reported yet.

It has been shown by studies in the literature that the enzyme CYP2E1 which mediates the hepatocyte damage caused by a variety of compounds, such as ethanol and CCl_4_, is considered to be an important determinant of human susceptibility to toxicity and carcinogenicity caused by the industrial and environmental chemicals [15]. CYP3A4, a major enzyme that is expressed in adult liver, has been considered to be an important factor affecting drug absorption. CYP2E1 and CYP3A4 are not only the metabolic enzymes directly involved in hepatic oxidative damage, but they also are two important targets of the oxidation mechanism of acute liver injury [16]. Quercetin, kaempferol, isoquercitrin, astragalin and other flavonoids have strong antioxidant activities [9,17], therefore, we speculated that the flavonoids might be active compounds that can reduce the contents of liver-damaging substances by inhibiting the activities of CYP450 enzymes, which might be one of the hepatoprotective mechanisms of *L. clethroides*.

On the basis of previous studies, the impacts of quercetin, kaempferol, isoquercitrin and astragalin on the activities of enzymes CYP2E1 and CYP3A4 in rat liver microsomes were examined, aiming to investigate the correlation between the hepatoprotective effects of *L. clethroides* and the antioxidant activities of these four compounds. These studies could provide an important basis for the development of *L. clethroides* as a liver-protective drug and guide the rational use of these drugs in the clinic.

## 2. Results

### 2.1. Method Validation

#### 2.1.1. Standard Curves and the Linearity

Different concentrations of the standard solutions mentioned in Section 4.6 were accurately injected into the chromatographic instrument for the construction of calibration curves. Then the standard curves were constructed by plotting the ratios of peak areas of metabolites and phenacetin (internal standard) *versus* the concentrations of their metabolites. The results are listed in Table 1. Within the selected concentration ranges, good linearities were obtained, with *r* values of 0.9999 and 0.9998, respectively.

#### 2.1.2. Precision and Stability

The precision and stability of the used methods were determined by the relative standard deviation (RSD). The precision was obtained by analyzing the standard solutions at three different concentrations six consecutive times, respectively. The stability was obtained by analyzing the standard solutions at three different concentrations six times in one day. Every experiment was performed in triplicate. The results are shown in Table 1.

#### 2.1.3. Recovery

Different concentrations of 4-nitrocatechol (at final concentrations of 5, 10 and 15 μM) and 6β-hydroxytestosterone (final concentrations of 0.15, 0.30 and 0.45 μg·mL^−1^) were added to the incubation systems *in vitro*, respectively. Then spiked samples were prepared and measured according to the methods described in Section 4.5. The results, presented in Table 2, demonstrate that the methods were reasonable and feasible.

### 2.2. The Optimal Conditions of Incubation System in Vitro

**Figure 1:**
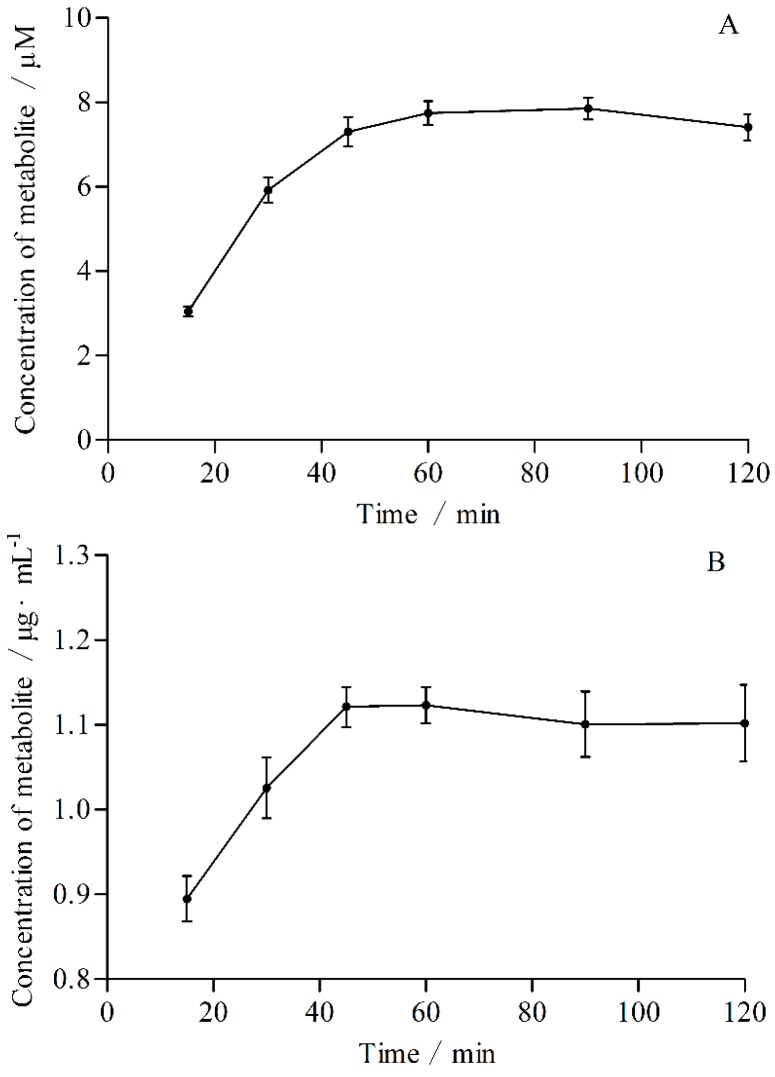
Influences of incubation times on the production rates of 4-nitrocatechol (**A**) and 6β-hydroxytestosterone (**B**).

**Figure 2:**
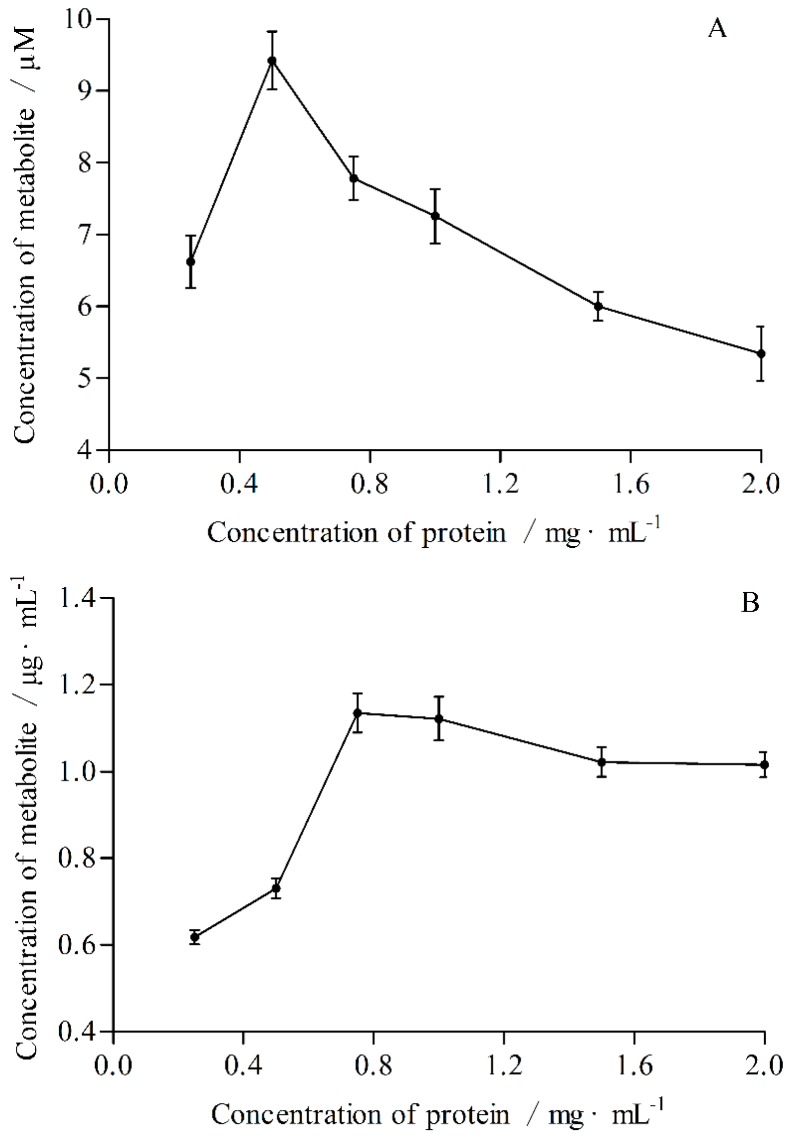
Influences of protein concentrations on the production rates of 4-nitrocatechol (**A**) and 6β-hydroxytestosterone (**B**).

The optimal incubation times and protein concentrations were determined according to the linear relationship between incubation time or concentration of protein and concentration of metabolite, respectively (Figure 1 and Figure 2). The optimal incubation times for CYP2E1 and CYP3A4 were 60 and 45 min, respectively. The optimal protein concentrations for CYP2E1 and CYP3A4 were 0.5 and 0.75 mg·mL^−1^, respectively.

**Figure 3:**
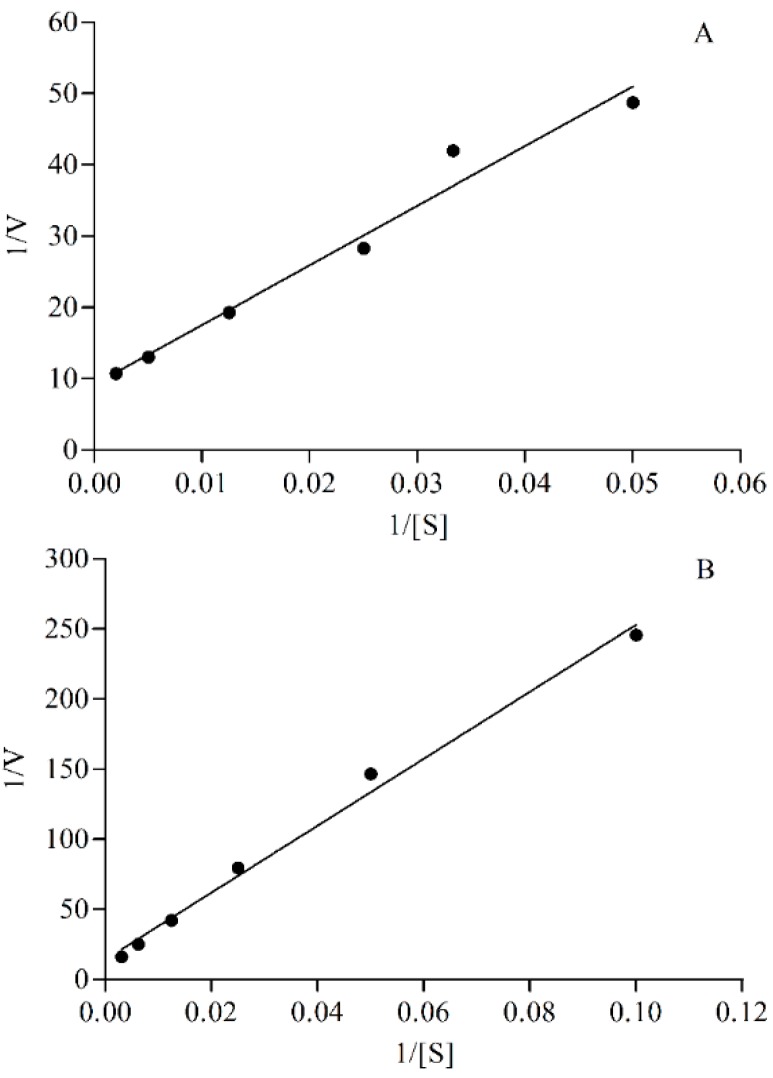
Lineweaver-Burk plot of 4-nitrocatechol (**A**) and 6β-hydroxytestosterone (**B**).

The apparent K_m_ (Michaelis constant) value was calculated according to Michaelis-Menten equation and Lineweaver-Burk plots (Figure 3). The regressive equations obtained by the Lineweaver-Burk method for CYP2E1 and CYP3A4 were *Y* = 835.9883*X* + 9.1943 (*r* = 0.9864) and *Y* = 2388.0110*X* + 14.1598 (*r* = 0.9961) respetively. The K_m_ values calculated were 90.92 and 168.65 μM, respectively. The concentration of probe substrate in incubation system should not exceed the K_m_ values [18], thus, 90 and 160 μM for 4-nitrophenol and testosterone were chosen in this study.

### 2.3. Effects of Flavonoids in L. clethroides on CYP450 Activity

#### 2.3.1. Effects on CYP2E1 Activity

**Figure 4:**
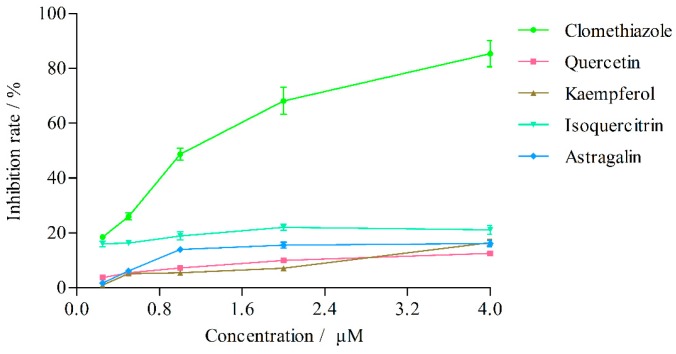
Effects of flavonoids on CYP2E1 isozyme activities.

**Figure 5:**
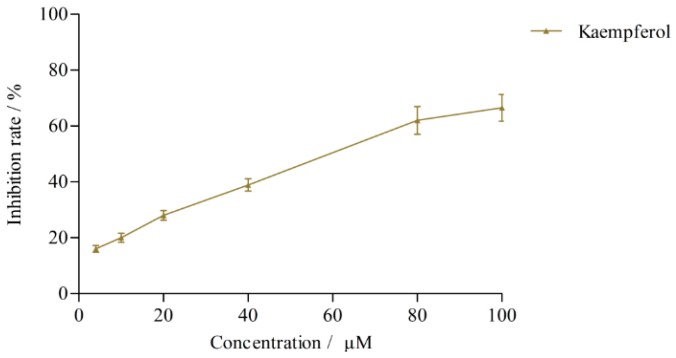
Effect of kaempferol on CYP2E1 isozyme activity.

The effects of quercetin, kaempferol, isoquercitrin and astragalin in *L. clethroides* and clomethiazole (positive control) on CYP2E1 activity are shown in Table 3 and Figure 4. Clomethiazole inhibited the production of 4-nitrocatechol with an IC_50_ of 1.07 ± 0.01 μM. The effects of quercetin, kaempferol, isoquercitrin and astragalin on CYP2E1 acivity were very weak and their IC_50_ values could not be calculated. When its concentration was gradually increased to 100 μM [19], kaempferol inhibited the production of 4-nitrocatechol with an IC_50_ of 60.26 ± 2.54 μM (Figure 5), but at this concentration, the other three flavonoids still could not cause 50% inhibition of the enzyme activity.

#### 2.3.2. Effects on CYP3A4 Activity

**Figure 6:**
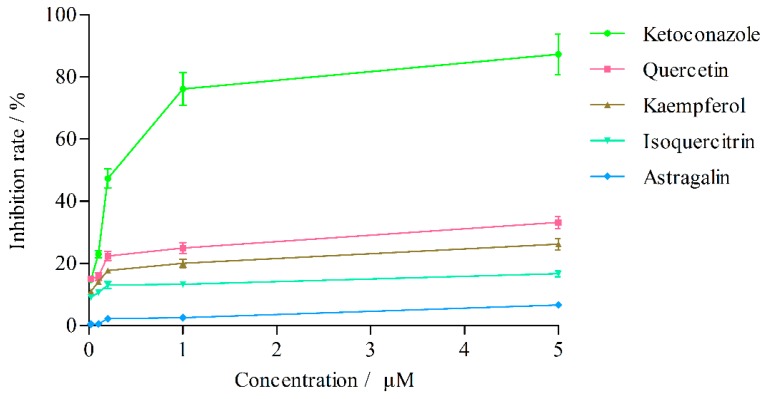
Effects of flavonoids on CYP3A4 isozyme activities.

**Figure 7:**
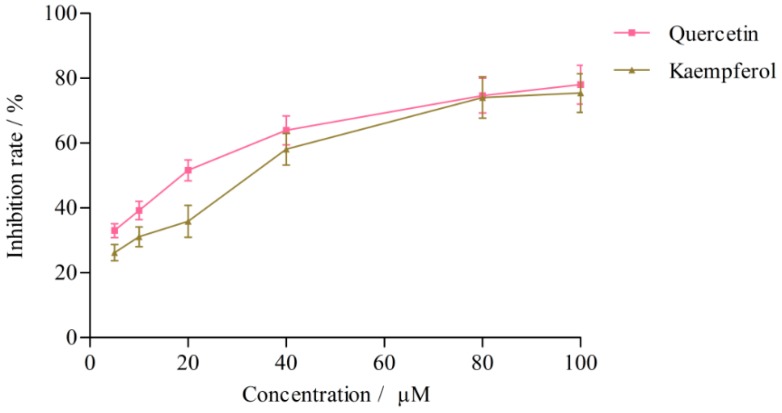
Effects of quercetin and kaempferol on CYP3A4 isozyme activities.

The effects of quercetin, kaempferol, isoquercitrin and astragalin in *L. clethroides* and ketoconazole (positive control) on CYP3A4 activity are shown in Table 4 and Figure 6. Ketoconazole strongly inhibited the production of 6β-hydroxytestosterone (IC_50_ = 0.25 ± 0.01 μM), but the effects of the four flavonoids in *L. clethroides* on CYP3A4 were very weak and their IC_50_ values could not be calculated. When the concentrations of the four flavonoids were gradually increased to 100 μM, quercetin and kaempferol could inhibit the production of 6β-hydroxytestosterone with IC_50_ values of 18.77 ± 1.69 μM and 32.65 ± 1.32 μM, respectively (Figure 7).

## 3. Discussion

CYP2E1 and CYP3A4 are two important metabolic enzymes involved in liver injury. Total alkaloids of *Rubus alceaefolius* Poir could significantly reduce the levels of GOT and GPT, protect liver cells from injury, and inhibit the mRNA expressions of CYP2E1 and CYP3A1 in liver tissue [20]. It has been indicated that Gegen powder possesses therapeutic effects on acute alcohol-induced liver injury, by increasing the content of CYP450 and reducing the activity of CYP2E1 [21]. *Radix Glycyrrhizae* could protect the liver injury caused by *Rhizoma dioscorea* bulbifera, possibly due to its induction of activity of CYP2E1 and CYP3A4 and inhibition of the mRNA expression [22].

Clomethiazole and ketoconazole act as the positive inhibitors for CYP2E1 and CYP3A4 can significantly inhibit the formation of metabolites. The IC_50_ values of clomethiazole and ketoconazole were 1.07 ± 0.01 μM and 0.25 ± 0.01 μM, respectively, which are consistent with literature values [23,24], suggesting that the incubation systems *in vitro* can meet the activities of measurement of CYP2E1 and CYP3A4.

According to the literature [25,26], a strong inhibition is considered for a compound with an IC_50_ value below 1 μM, and if the IC_50_ value is higher than 50 μM, the compound is considered to be a weak inhibitor. Thus, kaempferol has a weakly inhibitory effect on CYP2E1, while quercetin and kaempferol have moderate inhibitory effects on CYP3A4.

The results of this study showed that the two flavone glycosides (isoquercitrin and astragalin) had no effect on the activities of CYP2E1 and CYP3A4. This might be due to the reason that there is a certain quantitative structure-activity relationship between the structure of the flavones and their bioactivities. A previous study had shown that the number and location of flavonoids’ hydroxy substituents and the conjugated system all have significant influence on the activities of CYP450 enzymes [27]. As the number of hydroxy substitutions was increased, the inhibition of CYP450 enzymes activities tended to be progressively enhanced. The high polarity of the glycosides may also have interfered with their interaction with the CYP450 enzymes [28]. Currently, few studies have been done on the structure of the compounds and their inhibition effects and further studies are needed.

Cytochromes P450, especially CYP2E1 and CYP3A4, are responsible for metabolism of ethanol, CCl_4_ and other solvents in the body. In the model of alcohol-induced liver injury, the expression levels of CYP2E1 and CYP3A4 were enhanced [29]. Researchers have used CYP2E1 knockout mice to evaluate the effect of CYP2E1 on ethanol-induced chronic liver injury, and the results showed that the fatty liver production and ethanol-induced oxidative stress of wild-type mice were significantly higher than those in CYP2E1 knockout mice [30], showing that CYP2E1 is involved in the oxidative stress of the body, increasing the severity of liver damage.

As a typical poison, CC1_4_ is activated by CYP2E1 and metabolized to a series of highly reactive free radicals. These radicals can attack the cell membrane by hydrogen adsorption and induce lipid peroxidation. The cellular calcium concentration is augmented along with radical reaction-induced cell death [31]. Lipid peroxidation alters the integrity of cellular membranes and damages proteins and DNA, decreases hepatic antioxidants such as glutathione (GSH), finally lead to hepatic oxidative damage even canceration [32]. 

The occurrence of liver injury can be reduced or even suppressed by minimizing the activities of CYP2E1 and CYP3A4, making the discovery of more compounds with anti-oxidative stress from natural products of important significance.

## 4. Material and Methods

### 4.1. Chemicals

Chromatographic grade acetonitrile was purchased from Avantor Performance Materials, Inc. (Center Valley, PA, USA). Chromatographic grade methanol was purchased from Tianjin Shield Fine Chemicals Co., Ltd. (Tianjin, China) Analytical grade glacial acetic acid was purchased from Tianjin Fuyu Fine Chemical Co., Ltd. (Tianjin, China) The water was Wahaha pure water. Coomassie brilliant blue was purchased from Nanjing Jiancheng Bioengineering Institute (Nanjing, China). The isoquercitrin, astragalin, quercetin and kaempferol with purity greater than 98% were purchased from Chengdu Pufei De Biotech Co., Ltd. (Sichuan, China). The 4-nitrocatechol and 4-nitrophenol were purchased from A Johnson Matthey Company (Royston, UK). Phenacetin and 6β-Hydroxytestosterone and clomethiazolewere purchased from Sigma (St. Louis, MO, USA). Testosterone was purchased from Beijing J & K Technology Co., Ltd. (Beijing, China). NADPH was purchased from Blue Chemical Technology Co., Ltd. (Shanghai, China). Ketoconazole was purchased from Tokyo Chemical Industry (Tokyo, Japan).

### 4.2. Instruments

A LC-20AT high performance liquid chromatography system (Shimadzu, Kyoto, Japan), equipped with a degasser, a quaternary gradient low pressure pump, the CTO-20A column oven, a SPD-M20A UV-detector, a SIL-20A autosampler was used. The data were acquired and processed using a LC-Solution chromatography data processing system. Chromatographic separations of target analytes were performed on an InertSustain C18 column (4.6 mm × 150 mm, 5 μm). A GRP-9270 water-jacket thermostatic constant incubator purchased from Shanghai Sumsung Laboratory Instrument Co., Ltd. (Shanghai, China) was used. A TGL-16gR high-speed freezing centrifuge was purchased from Shanghai Anting Scientific Instrument Factory (Shanghai, China). The Power Gen 125 tissue homogenizing machine was purchased from Fisher Scientific (Waltham, MA, USA).

### 4.3. Animals

Sprague Dawley (SD) rats (200–220 g) were obtained from the Experimental Animal Center of Henan Province (Zhengzhou, Hennan, China). The animals were kept under standard conditions (12 h light/dark cycle, 25 °C and humidity 50% to 65%) and housed in polycarbonate cages with standard rodent diet and water. All the animal procedures were approved by the local ethical committee in accordance with the “Institute Ethical Committee Guidelines” for animal experimentation and care (HNPR-2009-05003).

### 4.4. Preparation of Rat Liver Microsomes 

The rats were fasted for 12 h with free access to water and then sacrificed by cervical dislocation. The liver was removed promptly and washed with ice-cold physiological saline solution to yellowish brown. The liver tissue was cut into pieces as small as possible and the liver homogenate solution of 25% with phosphate buffer (pH 7.4) was processed at 0 to 4 °C by a tissue homogenizing machine. The liver homogenate solution was centrifuged at 4 °C for 20 min at 12,000 r·min^−1^ and supernatant fraction was reserved. The supernatant per millilitre was added with 0.1 mL CaCl_2_ (88 mM), gently shaken and kept at 0 to 4 °C for 5 min. The mixed solution was transferred to centrifuge tubes and centrifuged at 4 °C for 30 min at 15,000 r·min^−1^. Then the supernatant was removed completely, the precipitation was re-suspended with moderate phosphate buffer and centrifuged at 4 °C for 30 min at 15,000 r·min^−1^ [33,34]. The pink precipitate obtained after centrifugation was used as the liver microsomes. The liver microsomes was resuspended in moderate phosphate buffer containing 20% glycerol and divided into two parts. One part was packed and stored at −80 °C for further analysis, and another part was used for determination of protein concentration. Protein concentration of the microsomes was determined by the method of Bradford.

### 4.5. Cytochrome P450 Probe Substrate Assays

#### 4.5.1. 4-Nitrophenol 2-Hydroxylation Assay for CYP2E1

The *in vitro* incubation system of CYP2E1 contained liver microsomes, MgCl_2_ (5 mM), 4-nitrophenol (probe substrate) and phosphate buffer (pH 7.4) in a final volume of 200 μL. The mixture was pre-incubated 5 min at 37 °C thermostatic constant incubator, the reaction was initiated by adding NADPH (1 mM). After incubation, 100 μL of ice-cold acetonitrile containing 50 μM phenacetin was added to the incubation system (phenacetin was used as internal standard). After blending, the mixture was kept at ice-water bath to terminate the reaction, and then centrifuged at 4 °C for 20 min at 12,000 r·min^−1^. Then, the supernatant was filtrated through a 0.22 μm microporous membrane and the subsequent filtrate was collected. The concentration of organic solvent was not higher than 1%.

**Figure 8:**
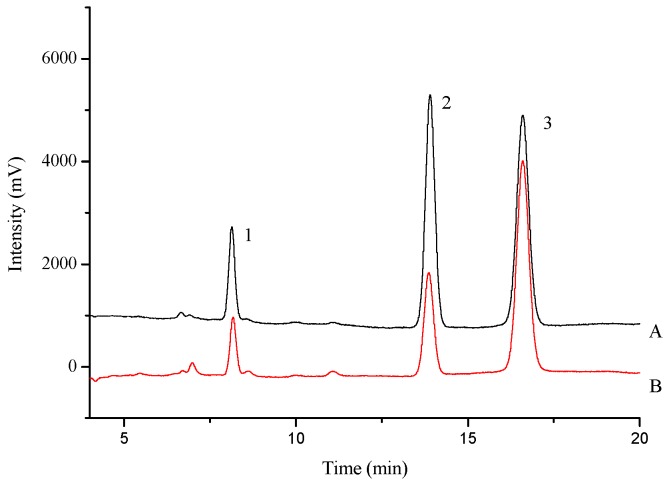
HPLC chromatograms of standard solution (**A**) and sample solution (**B**) 1: 4-nitrocatechol; 2: 4-nitrophenol; 3: phenacetin.

The mobile phase of chromatographic separation consisted of methanol and 0.1% acetic acid (33:67, *v*/*v*), at a flow rate of 1.0 mL·min^−1^. The column temperature was set to 30 °C. The UV detection wavelength was set at 250 nm. All the injection volumes were 10 μL. HPLC chromatograms of the standard solution and sample are shown in Figure 8.

#### 4.5.2. Testosterone 6β-Hydroxylation Assay for CYP3A4

**Figure 9:**
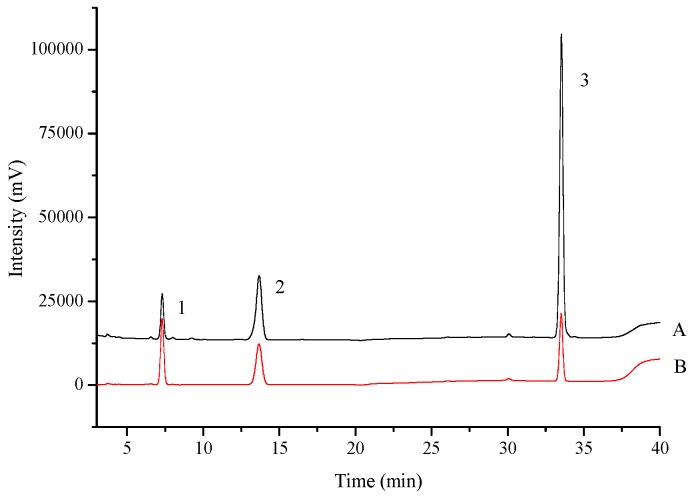
HPLC chromatograms of standard solution (**A**) and sample solution (**B**) 1: phenacetin; 2: 6β-hydroxytestosterone; 3: testosterone.

The incubation conditions of CYP3A4 were basically the same as those described in Section 4.5.1 but the volume of incubation mixture was 500 μL and the reactions were terminated by adding 700 μL of ice-cold acetonitrile containing 25 μM phenacetin. The mobile phase for separating testosterone, 6β-hydroxytestosterone and phenacetin consisted of acetonitrile (A), methanol (B) and 0.1% acetic acid (C), and the gradient program was set as follows: 0–15 min, 45% B, 55% C; 15–25 min, 0%–35% A, 45%–10% B, 55% C; 25–40 min, 35% A, 10% B, 55% C. The flow rate was 1.0 mL·min^−1^. The column temperature was set to 30 °C. The UV detection wavelength was set at 245 nm. All the injection volumes were 20 μL. HPLC chromatograms of the standard solution and sample are shown in Figure 9.

### 4.6. Preparation of Standard Solutions

Different concentrations of 4-nitrocatechol (0.8–51.2 μM) and 6β-hydroxytestosterone (0.1–25.6 μg·mL^−1^), respectively, were added *in vitro* to the incubation systems. Other operational procedures were basically the same as those described in Section 4.5, but the NADPH in the incubation system was replaced with an equal volume of phosphate buffer.

### 4.7. Optimization of Incubation Conditions in Vitro

#### 4.7.1. Incubation Time

The concentrations of protein and probe substrate of incubation system were fixed, the incubation times were 15, 30, 45, 60, 90 and 120 min, respectively. Samples were preparated according to the methods described in Section 4.5. The concentrations of probe substrate metabolites 4-nitrocatechol and 6β-hydroxytestosterone were calculated by referring to standard curves. The optimal incubation times were determined according to the linear relationship between concentration of metabolite and time.

#### 4.7.2. The Concentration of Protein

The incubation time and probe substrate concentration of incubation system were constant, the protein concentrations were 0.25, 0.5, 0.75, 1.0, 1.5 and 2.0 mg·mL^−1^, respectively. Samples were preparated and the metabolite concentrations were determined according to the methods described in Section 4.5. The optimal concentrations of protein were determined by the linear relationship between concentration of metabolite and protein.

#### 4.7.3. The Concentration of Probe Substrate

A series of concentrations of 4-nitrophenol (20–500 μM) and testosterone (10–320 μM) were added to the incubation system in order to determine the optimal concentrations of probe substrates *in vitro*. The apparent K_m_ (Michaelis constant) value was calculated according to the Michaelis-Menten equation and Lineweaver-Burk plot.

### 4.8. Effects of Flavonoids in L. clethroides on CYP450 Activity

#### 4.8.1. CYP2E1 Assay

To evaluate whether quercetin, kaempferol, isoquercitrin and astragalin affect the activity of CYP2E1, the studies were carried out in three groups. Group 1 (normal control) was added with phosphate buffer, group 2 (flavonoids reagent group) were added with flavonoids, and group 3 was given clomethiazole as positive control. Groups 2 and 3 experiments were performed at concentrations of 0.25, 0.5, 1, 2 and 4 μM, and all the the experiments were performed in triplicate. The concentrations of probe substrate metabolites were calculated from the standard curves. The percentage of inhibition was calculated using the formula: %Inhibition = ((A_0_ − A_1_)/A_0_) × 100%, in which A_0_ was the concentration of probe substrate metabolite of group 1 and A_1_ was the concentration of probe substrate metabolite of group 2 and 3. The IC_50_ values were calculated based on the concentration-effect linear regression curve.

#### 4.8.2. CYP3A4 Assay

The effects of flavonoids in *L. clethroides* on CYP3A4 activity were basically the same as CYP2E1. The positive control was ketoconazole, and Groups 2 and 3 experiments were performed at concentrations of 0.02, 0.1, 0.2, 1 and 5 μM [35].

## 5. Conclusions

*In vitro* rat liver microsomes incubation assay methods were adopted for determining the effects of quercetin, kaempferol, isoquercitrin and astragalin in *L. clethroides* on the activities of CYP2E1 and CYP3A4. In conclusion, kaempferol has a weakly inhibitory effect on CYP2E1, while quercetin and kaempferol had moderate inhibitory effects on CYP3A4 activity. Based on these results, it can be speculated that the active hepatoprotective ingredients of *L. clethroides* include quercetin and kaempferol, which confer the inhibitory effects on the activities of CYP2E1 and CYP3A4. The reduction of CYP2E1 and CYP3A4 activities can mitigate the biotransformation of CC1_4_ and prevent the production of liver damaging substances to exert a liver protective role. Of course, the *in vivo* and *vitro* activities may be different, a further verification of the above speculation needs to be done by *in vivo* experiments.

## Figures and Tables

**Table 1:** Analytical performances.

Metabolite		4-Nitrocatechol	6β-Hydroxytestosterone
Regressive equation		*Y* = 0.0184*X* − 0.0042	*Y* = 0.0880*X* − 0.0062
*r*		0.9999	0.9998
Linear range		0.8–51.2 μM	0.1–25.6 μg·mL^−1^
Precision (RSD, %)	High concentration	0.25	0.22
	Moderate concentration	0.35	0.46
	Low concentration	3.93	0.96
Stability (RSD, %)	High concentration	0.28	0.075
	Moderate concentration	0.80	0.17
	Low concentration	2.63	1.39

**Table 2:** Recoveries of the metabolites.

Metabolite	Original	Added	Found	Recovery (%)	RSD (%)
4-Nitrocatechol (μM)	10.9	5	15.4 ± 0.11	89.4 ± 2.27	5.02
	10.9	10	19.7 ± 0.09	87.1 ± 0.85	
	10.9	15	25.5 ± 0.17	96.8 ± 1.12	
6β-Hydroxytestosterone (μg·mL^−1^)	0.296	0.15	0.43 ± 0.01	91.5 ± 4.77	4.40
	0.296	0.30	0.55 ± 0.01	85.4 ± 1.01	
	0.296	0.45	0.69 ± 0.01	86.9 ± 2.46	

**Table 3:** Effects of flavonoids on CYP2E1 isozyme activities (*x* ± *s, n* = 3).

Concentration (μM)	Inhibition Rate (%)				
	Clomethiazole	Quercetin	Kaempferol	Isoquercitrin	Astragalin
4	85.41 ± 4.78	12.54 ± 0.81	16.49 ± 1.23	21.12 ± 1.56	16.16 ± 1.13
2	68.15 ± 4.97	9.99 ± 0.71	7.12 ± 0.50	22.06 ± 1.12	15.58 ± 1.02
1	48.70 ± 2.19	7.21 ± 0.43	5.50 ± 0.29	18.91 ± 1.43	13.97 ± 0.81
0.5	25.99 ± 1.27	5.46 ± 0.33	5.09 ± 0.37	16.27 ± 0.83	6.14 ± 0.45
0.25	18.49 ± 0.59	3.68 ± 0.30	0.97 ± 0.80	16.02 ± 1.05	1.73 ± 0.13

**Table 4:** Effects of flavonoids on CYP3A4 isozyme activities (*x* ± *s, n* = 3).

Concentration (μM)	Inhibition Rate (%)				
	Ketoconazole	Quercetin	Kaempferol	Isoquercitrin	Astragalin
5	87.28 ± 6.55	33.18 ± 1.96	26.20 ± 1.81	16.68 ± 1.00	6.65 ± 0.41
1	76.11 ± 5.25	24.97 ± 1.77	20.00 ± 1.28	13.31 ± 0.63	2.57 ± 0.19
0.2	47.33 ± 3.08	22.41 ± 1.46	17.70 ± 0.80	13.04 ± 1.11	2.22 ± 0.14
0.1	22.95 ± 1.08	15.79 ± 1.23	14.12 ± 0.55	10.62 ± 0.62	0.51 ± 0.04
0.02	14.72 ± 0.56	15.04 ± 0.83	10.98 ± 0.87	9.03 ± 0.35	0.42 ± 0.04
